# Suggestive Therapeutics in Psychopathia Sexualis

**Published:** 1895-05

**Authors:** 


Suggestive Therapeutics in Psychopathia Sexualis. With especial reference to contrary sexual instinct. By Dr. A. von Schrenck-Notzing, Munich, Germany. Authorized translation from the German by Charles Gilbert Chaddock, M. D., Professor of Diseases of the Nervous System, Marion-Sims College of Medicine, St. Louis; member of the American Medico-Psychological Association; Attending Neurologist to the Rebekah Hospital, St. Louis, Mo., etc., etc. One volume, royal octavo, 325 pages. Extra Cloth, $2.50 net; Sheep, $3.50 net. Sold only by subscription to the medical profession exclusively. Philadelphia: The F. A. Davis Co., Publishers, 1914 and 1916 Cherry Street.

Less than two years ago was published a book entitled *Psychopathia Sexualis*, by Dr. R. von Krafft-Ebing. This book was reviewed at length in The Homœopathic Physician for September, 1893, at page 478.

The present volume under review is a sort of sequel or we may say a companion to the other. It deals with the same subjects as the book of Krafft-Ebing and aims at effecting a cure.

The author points out the necessity of individualization at page 42. He advises careful hygienic surroundings, cold baths, electricity, and drugs.

But his main reliance in treatment is upon psychical treatment. He seeks to induce self-knowledge and self-control, and these are supplemented by his main reliance and we might say, "trump card"—"hypnotic suggestion."

All the means of treatment used he classifies as medical, mechanical, electrical, religious, and mystical.

His principal method as before stated is hypnotic suggestion.

This consists mainly in putting the patient under the influence of hypnotism, in which state suggestions in the direction of healthy, natural, normal exercise of the functions of procreation are infused into the patient's mind, and then the hypnotic condition is removed. Under such circumstances the patient improves, the objectionable ideas fade away and after a number of repetitions of the treatment the patient is cured.

Many instances are related and much valuable information analyzing the conditions of the various sexual anomalies is given.

Having no especial experience with hypnotic suggestion, the editor of this journal is not competent to give an opinion upon this method of dealing with these horrible practices.

This book, as well as its original, *Psychopathia Sexualis*, before referred to, is of great value, because of the new light it throws on these subjects.

The author claims to cure by this method sixty-five per cent. of the onanists whom he has treated.

It is to be understood that in treating these people, account is taken also of all psychological principles that will in any way contribute a salutary influence toward cure.

Thus the author says, at page 96, "Psychotherapeutics takes account of the patient's judgment, the co-ordinating cerebral activity, and sets in motion his will, his attention, and his judgment. Suggestions, on the contrary, have the special tendency to cause the idea imparted to be transformed into an act." Thus it will appear that in order to treat such cases intelligently, the physician must have a wide acquaintance with the phenomena of the mind. This knowledge the author of the book under notice undoubtedly possesses and it is abundantly set forth in its pages.

